# Supplementary material for: Splice-Junction-Based Mapping of Alternative Isoforms in the Human Proteome
Source: Cell Rep. Author manuscript; Available in PMC 2020 Jan 15. (PMC6961840; doi:10.1016/j.celrep.2019.11.026)
Supplement: 3 [file NIHMS1546469-supplement-3.zip › DF2/PXD006675/RightVentricle-189-P52179-AAIAPPSPPCDITCLESFRDSMVLGWK.pdf]

A

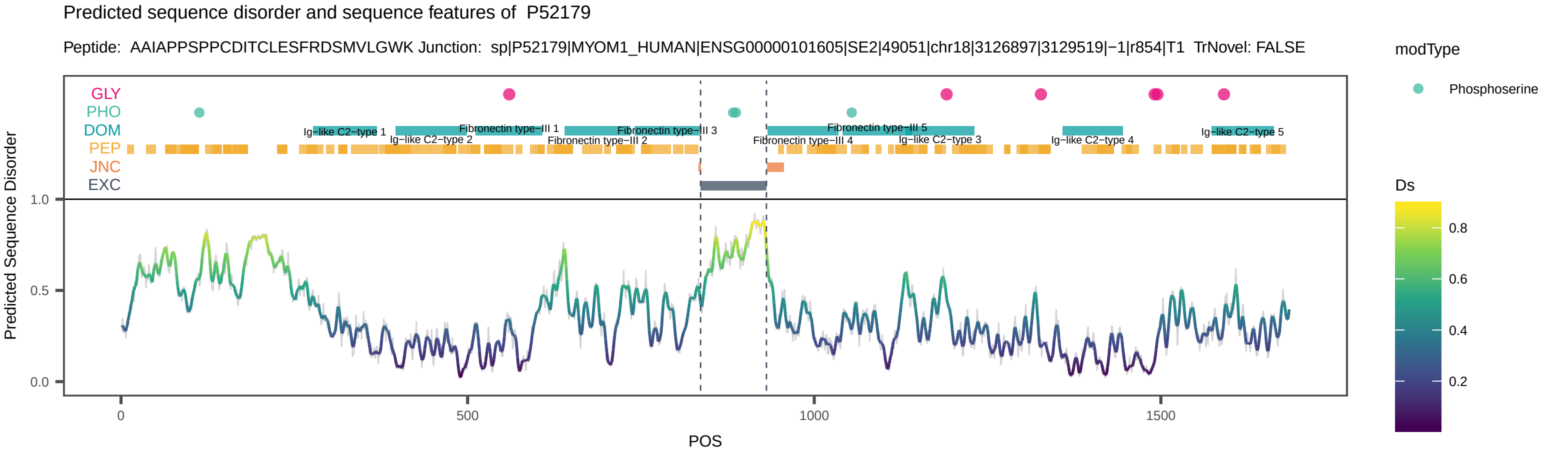

Predicted sequence disorder and sequence features of P52179
Peptide: AAIAPPSPPCDITCLESFRDSMVLGWK Junction: sp|P52179|MYOM1_HUMAN|ENSG00000101605|SE2|49051|chr18|3126897|3129519|−1|r854|T1 TrNovel: FALSE
GLY
PHO
DOM
PEP
JNC
EXC
Ig−like C2−type 1
Ig−like C2−type 2
Fibronectin type−III 1
Fibronectin type−III 2
Fibronectin type−III 3
Fibronectin type−III 4
Fibronectin type−III 5
Ig−like C2−type 3
Ig−like C2−type 4
Ig−like C2−type 5
Predicted Sequence Disorder
1.0
0.5
0.0
0
500
1000
1500
POS
modType
Phosphoserine
Ds
0.8
0.6
0.4
0.2

B

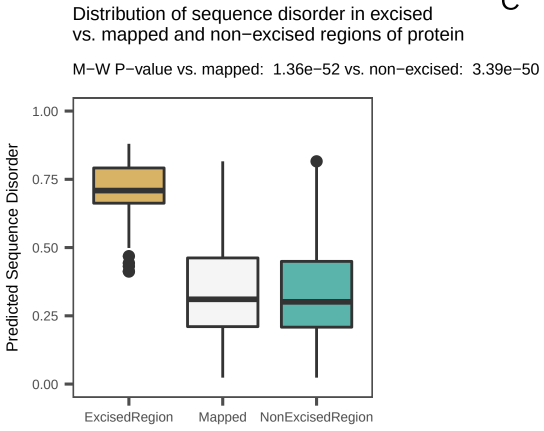

Distribution of sequence disorder in excised
vs. mapped and non−excised regions of protein
M−W P−value vs. mapped: 1.36e−52 vs. non−excised: 3.39e−50
Predicted Sequence Disorder
1.00
0.75
0.50
0.25
0.00
ExcisedRegion
Mapped
NonExcisedRegion

C

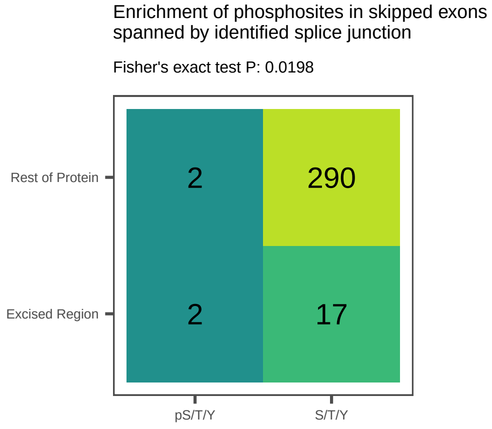

Enrichment of phosphosites in skipped exons
spanned by identified splice junction
Fisher's exact test P: 0.0198
Rest of Protein
Excised Region
2
290
2
17
pS/T/Y
S/T/Y
